# Task shifting of HIV/AIDS case management to Community Health Service Centers in urban China: a qualitative policy analysis

**DOI:** 10.1186/s12913-015-0924-y

**Published:** 2015-07-02

**Authors:** Fuchang Ma, Fan Lv, Peng Xu, Dapeng Zhang, Sining Meng, Lahong Ju, Huihui Jiang, Liping Ma, Jiangping Sun, Zunyou Wu

**Affiliations:** National Center for AIDS/STD Control and Prevention, Chinese Center for Disease Control and Prevention, 155 Changbai Road, Changping District Beijing, 102206 China

**Keywords:** Task shifting, Community Health Service Center, Policy analysis, HIV/AIDS

## Abstract

**Background:**

The growing number of people living with HIV/AIDS (PLWHA) in China points to an increased need for case management services of HIV/AIDS. This study sought to explore the challenges and enablers in shifting the HIV/AIDS case management services from Centers for Disease Control and Prevention (CDCs) to Community Health Service Centers (CHSCs) in urban China.

**Methods:**

A qualitative method based on the Health Policy Triangle (HPT) framework was employed to gain in-depth insights into four elements of the task shifting strategy. This included a review on published literature and health policy documents, 15 focus group discussions (FGDs) and 30 in-depth interviews (IDIs) with four types of key actors from three cities in China. A total of 78 studies and 17 policy files at the national, municipal and local levels were obtained and reviewed comprehensively. Three semi-structured interview guides were used to explore key actors’ views on shifting the HIV/AIDS case management services to CHSCs.

**Results:**

It is necessary and feasible for CHSCs to engage in case management services for PLWHA in local communities. The increasing number of PLWHA and shortage of qualified health professionals in CDCs made shifting case management services downwards to CHSCs an urgent agenda. CHSCs’ wide distribution, technical capacity, accessibility and current practice enabled them to carry out case management services for PLWHA. However our findings indicated several challenges in this task shifting process. Those challenges included lack of specific policy and stable financial support for CHSCs, inadequate manpower, relatively low capacity for health service delivery, lack of coordination among sectors, PLWHA’s fear for discrimination and privacy disclosure in local communities, which may compromise the effectiveness and sustainability of those services.

**Conclusions:**

Shifting the HIV/AIDS case management services from CDCs to CHSCs is a new approach to cope with the rising number of PLWHA in China, but it should be implemented alongside with other efforts and resources such as increasing public funding, planned team building, professional training, coordination with other sectors and education on privacy protection as well as non-discrimination to make this approach more effective and sustainable. Policy makers need to ensure both political feasibility and resources accessibility to facilitate this shifting process.

## Background

The number of people living with HIV/AIDS (PLWHA) was estimated 780 000 in China as of 2011, among which approximately 154 000 had developed AIDS [1]. Since the launch of the national free Antiretroviral Treatment (ART) program in 2003, China has achieved impressive ART coverage. By the end of 2011, a total of 122 613 individuals had been receiving ART, accounting for 79.6 % of the total estimated eligible AIDS cases [2–4]. HIV/AIDS case management, which refers to regular follow-up intervention services, plays a key role in improving adherence to ART and reducing risk behaviors. In China, these services are usually carried out by health professionals from Centers for Disease Control and Prevention (CDCs) at the municipal, district and county levels throughout the country [5, 6].

However, as the reported number of PLWHA increased partially due to the scaling up of HIV testing and ART programs in recent years [7], the expanding workload and shortage of human resources in local CDCs have emerged as new challenges and compromised the quality of HIV/AIDs case management services in recent years [8, 9].To respond to these challenges, task shifting approach recommended by World Health Organization (WHO) can be applied to expand health care workforce and improve access to HIV/AIDS services rapidly. The task shifting approach is based on reorganization and decentralization of health care services, which may be particularly suitable in resource-constrained regions with high HIV/AIDS disease burden [10–12].

China has enhanced health care services for PLWHA in many rural areas with high HIV prevalence supported by the “Four Frees and One Care” program since 2004, which was particularly known as free voluntary counseling and testing and free ART drugs to AIDS patients who were rural residents or lived in urban areas without health insurance. In rural areas, local village doctors were assigned tasks such as health education and case management services. But this type of decentralization was not obvious in urban areas where HIV/AIDS preventive services were mostly administered by local CDCs [13–15]. Hence in urban areas, shifting a part of HIV/AIDS services from CDCs to Community Health Service Centers (CHSCs) has gained much attention both at the national and local level [16, 17]. CHSCs are essential components of China’s three-tier health delivery system providing basic medical and public health services in urban areas. Up till 2010, China has built 33 000 CHSCs in 625 cities nationwide with a total of 295 000 well-trained health providers [18, 19]. Current health policies such as the “Action Plan for HIV/AIDS prevention and Control in China (2010–2015)” and the “National Regulations on Basic Public Health Care (2011)” recommended CHSCs to involve in the HIV/AIDS preventive services. However these policies did not provide specific details or instructions on how CHSCs can fulfill the task of HIV/AIDS case management [20, 21]. In order to develop detailed strategies, we conducted this qualitative policy study to explore the challenges and enablers in the process of shifting the HIV/AIDS case management services from CDCs to CHSCs.

## Methods

### Conceptual framework

**Fig. 1:**
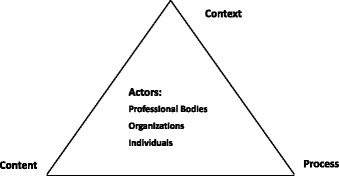
Policy analysis triangle (adapted from Walt and Gilson 1994). Detailed legends: The Policy analysis triangle consists of four elements: context (why need this policy), content (what is the policy mainly about), process (how was the policy brought forward and implemented) and actors (who participates in and influences formulation and implementation of the policy). In this study, actors mainly include officials and health professionals from different levels of health agencies, participants from community-based organizations and people living with HIV/AIDS (PLWHA) in local communities

The health policy triangle (HPT) framework was applied as the conceptual framework in this study. Developed by Walt and Gilson, this simplified approach helps researchers to understand and analyze health-related policies systematically [22, 23]. It consists of four elements: context (why), content(what), process(how) and actors (who) (See Fig. 1). Walt and Gilson believed that certain health policy-making was an interactive process within special social-economic and cultural context where actors were at the center of this process. In this study, the context of task shifting strategies was examined mainly from four dimensions of factors: situational factors, structural factors, technical factors and cultural factors [24]. HPT is a useful tool to explore different factors that might affect health policy and its implementation [25].

### Study sites and participants

This study was conducted in Guangzhou, Nanjing and Changsha city in 2013, all of which are capital cities of three provinces located in southeast of China. We selected four CHSCs in each city where a pilot program for comprehensive HIV/AIDS case management services was implemented at the time of study. Key actors were invited to attend focus group discussions (FGDs) and individual-based in-depth interviews (IDIs). Key actors were defined as those involved in provision, coordination or utilization of the aforementioned services and therefore could potentially influence relevant policies. We included four types of key actors: (1) 2 officials from the Bureau of Health (BH) and 27 health professionals from CDCs at the provincial, municipal and district levels; (2)37 administrators and health care providers from 12 CHSCs; (3)15 managers and volunteers from 8 local community-based organizations (CBOs); and (4)14 PLWHA in local communities. A purposive sampling method was used to select participants from the health agencies (BH, CDCs and CHSCs) and CBOs. PLWHA participants were recruited with assistance from the local CHSCs and CBOs. A total of 95 participants from three cities were included in the study. About two thirds of the participants from the health agencies had experiences in HIV/AIDS prevention services for more than one year (See Table 1).

**Table 1:** Socio-demographic characteristics of four categories of study participants

Characteristic	BH and CDCs	CHSCs	CBOs	PLWHA	Total
	No. (%)	No. (%)	No. (%)	No. (%)	No. (%)
City					
Guangzhou	14(48.3)	19(51.4)	4(26.7)	6(42.9)	43(45.3)
Nanjing	10(34.5)	9(24.3)	6(40.0)	6(42.9)	31(32.6)
Changsha	5(17.2)	9(24.3)	5(33.3)	2(14.2)	21(22.1)
Gender					
Male	16(55.2)	9(24.3)	12(80.0)	10(71.4)	47(49.5)
Female	13(44.8)	28(75.7)	3(20.0)	4(28.6)	48(50.5)
Age					
18–30	9(31.1)	11(29.7)	7(46.7)	3(21.4)	30(31.6)
31–45	17(58.6)	19(51.4)	5(33.3)	8(57.2)	49(51.6)
46 and above	3(10.3)	7(18.9)	3(20.0)	3(21.4)	16(16.8)
Education					
Middle/High school	0(0)	0(0)	1(6.7)	7(50.0)	8(8.4)
Associate college	0(0)	17(45.9)	0(0)	5(35.7)	22(23.2)
College and above	29(100.0)	20(54.1)	14(93.3)	2(14.3)	65(68.4)
HIV/AIDS service years					
1 and below	3(10.4)	18(48.6)	1(6.7)	---	22(27.2)
2–5	13(44.8)	13(35.2)	11(73.3)	---	37(45.8)
6 and above	13(44.8)	6(16.2)	3(20.0)	---	22(27.2)

*BH*, Bureau of Health; *CDCs*, Centers for Disease Control and Prevention; *CHSCs*, Community Health Service Centers; *CBOs*, Community-based organizations; *PLWHA*, People living with HIV/AIDS

### Data collection and analysis

We used a three-pronged approach in this study: (1) literature and health policy document review. A total of 78 studies and 17 policy files at the national, municipal and local levels were obtained and reviewed comprehensively, including strategic plans, regulations, technical guidelines and announcements regarding HIV/AIDS preventive services in CHSCs; (2) 15 FGDs with key actors from health agencies and CBOs were organized and conducted; (3) IDIs with 14 PLWHA and 16 participants from local CDCs, CHSCs and CBOs. Three semi-structured interview guides were applied to explore their views on shifting HIV/AIDS case management services from CDCs to CHSCs. In the FGDs and IDIs, we focused on four categories of issues: (1) challenges and enablers in the task shifting of HIV/AIDS case management services; (2) roles and views of different actors in shifting these services; (3) current practice of HIV/AIDS preventive services in CHSCs; and (4) acceptance of and access to case management services in CHSCs among PLWHA.

Both FGDs and IDIs were conducted in Chinese by experienced investigators for approximately 60–120 min in private rooms. All interviews were audio-taped except for some cases where only notes were taken at the request of the interviewees. Audiotapes were transcribed verbatim and the final transcripts were discussed among the investigators. All audio files and transcripts were anonymous. Using a qualitative content analysis method, information in the final transcripts were coded and placed in content tables under specific themes. Quotes, excerpts and summaries were extracted from the content tables and were discussed within the research team to reach a consensus. All transcriptions, coding and analyses were completed in Chinese and the results were translated into English. A narrative approach was used to summarize the data based on the HPT framework.

### Ethics statement

The participants were informed of the study purposes before the interviews and FGDs. All participants were voluntary and PLWHA respondents in the study received a cash reward for their participation and a travel expense reimbursement. However we didn’t obtain the consent for publishing the participants’ identification details. To protect their confidentiality, only two identifiers (sex and role) were presented after the quotes from the study participants. The study was approved by the Institutional Review Boards in the National Centers for AIDS/STD Control and Prevention (NCAIDS).

## Results

Data collected from three approaches were synthesized based on the HPT framework and particular attention was paid to the responses of key actors to the task shifting of HIV/AIDS case management services to CHSCs.

### Context of the task shifting

The context of shifting HIV/AIDS case management services to CHSCs was presented at four levels of factors (See Table 2). Currently, the increasing number of PLWHA and shortage of qualified health professionals in CDCs made shifting of case management services to CHSCs an urgent agenda. Meanwhile, the nationwide distribution of CHSCs with a large number of health care providers, broad coverage of the ART program, easily accessible skill training and promotion of anti-discrimination environment facilitated this task shifting in CHSCs in urban China.

**Table 2:** Context and process analysis on task shifting of HIV/AIDS case management services to CHSCs

Element	Description	Evidence
**Context**		
1. Situational factors	• A nationwide public health delivery system had been established defining CHSCs as the primary care institutions.	• Over 33 000 CHSCs and a total of about 300 000 trained employees around the country by 2010.
	• A large number of well-trained community doctors.	• An average of 100 CHSCs in each selected city.
2. Structural factors	• The number of PLWHA is increasing.	• The estimated and the reported number of PLWHA is continuously increasing in 2007, 2009 and 2011 in China.
	• The workload for HIV/AIDS prevention is expanding in CDCs.	
3. Technical factors	• CHSCs provide comprehensive medical and public health services including HIV/AIDS prevention and control.	• CHSCs serve as gate-keeper of health care delivery system in China.
	• The expansion of ART program in the country;	• Free ART became available for AIDS patients who were rural residents or urban residents with no health insurance since 2004.
4. Cultural factors	• The anti-discrimination campaign and human rights protection for PLWHA create supportive and legal environment for HIV/AIDS case management services in CHSCs.	• Issuance of the “Regulations on HIV/AIDS Prevention and Treatment” by the State Council in 2006.
		•The “Zero discrimination” goal and campaign in the society.
**Process**		
1. Agenda setting	• CHSCs need to participate in HIV/AIDS preventive services.	• China’s second Action Plan for the Containment and Control Of HIV/AIDS (2006–2010) by the State Council in 2006.
2. Policy development	• CHSCs need to assist upper level health institutions in HIV/AIDS health education and case management services.	• National Regulations on Basic Public Health Care by Ministry of Health in 2011.
	• CHSCs work as platform of China’s HIV/AIDS Care System.	• China’s Action Plan for the Containment and Control of HIV/AIDS (2011–2015) by the State Council in 2011.
	• Expansion the coverage of ART.	
	• HIV/AIDS case management services were included in the annual assessment of the basic public health services in three cities.	• The regulations or assessment announcements for community health service in Guangzhou, Nanjing and Changsha in 2012.
3. Implementation	• A pilot program integrating HIV/AIDS case management with routine health services in 42 CHSCs of eight cities (Beijing, Shanghai, Chongqing, Harbin, Nanjing, Hangzhou, Changsha and Guangzhou) was implemented from 2011 to 2013.	• China-Gates Foundation HIV Cooperation Program.
		• 77.6 % (1046/1348) of PLWHA have been receiving health management services in pilot CHSCs by the end of 2012 according to the program report.

*“There are more than 100 CHSCs in Nanjing city. We integrated HIV/AIDS case management into the basic public health services in CHSCs. This approach greatly improved the HIV/AIDS prevention and control in our city.” (Female, official from a municipal Bureau of Health)*

*“We have only two health professionals in our department engaging in all kinds of HIV/AIDS preventive services but have managed 300 PLWHA. Sometimes we seek help from other departments to finish the work.” (Female, health professional from a district CDC)*

### Process of the task shifting

Table 2 also showed main stages of the task shifting process. The State Council of China began to promote CHSCs’ participation in HIV/AIDS preventive services in 2006. A series of national policies were issued afterwards that guided CHSCs to assist or engage in HIV/AIDS case management services, which created an encouraging and legal environment for HIV/AIDS-related services in CHSCs.

Under the support of the China-Gates Foundation HIV/AIDS Cooperation Program, a pilot program which aimed at integrating HIV/AIDS case management with routine health care services has been initiated in 42 CHSCs of eight cities since 2011. Health care providers from those CHSCs were trained by the program experts following the national technical guideline. According to the program report, about 77.6 % of PLWHA have been receiving health management services in those CHSCs by the end of 2012. And with the promotion of this program, Guangzhou and Nanjing developed their own technical guidelines and regulations for delivery of case management services in CHSCs. All three cities in our study have included the HIV/AIDS case management service in the annual assessment of the basic public health services in CHSCs, which could influence their funding for each subsequent year.

### Content of the task shifting

As an integral part of a series of national and local HIV/AIDS policies, case management services in CHSCs aim to reduce HIV new infections as well as mortality rate, expand access to ART program and improve life quality of PLWHA. To achieve these goals, health care providers in CHSCs were required or encouraged to provide comprehensive case management services for PLWHA living in their communities with the support from local CDC and CBOs. The tasks were mainly included as follows:

1. Entering medical records: to enter medical records for all PLWHA and update their information regularly.
2. Follow-up interventions: to carry out intervention services for PLWHA, including counseling, psychological support and ART adherence education (twice for HIV-infected persons and four times for AIDS patients in a year, covering at least 85 % of PLWHA).
3. Testing: to provide CD4 count test for all PLWHA and HIV test for their HIV-negative spouses or regular sexual partners annually.
4. Treatment: to provide treatment for opportunistic infections, methadone maintenance treatment and ART for PLWHA with instruction from HIV/AIDS specialists.

### Key actors and their views on task shifting

Table 3 contains a summary of the key actors and their views on task shifting. Actors played different roles in the shifting process whose views were broadly positive or negative.

**Table 3:** Key actors and their views on task shifting of HIV/AIDS case management services to CHSCs

Actors	Roles in the task shifting	Positive Views	Negative Views
Officials and health professionals from BH and CDCs	Planning, organizing, supporting and evaluating the implementation of HIV/AIDS case management.	• It can improve the quality of services and effectiveness of case management.	• Lack of specific policy and financial support.
		• It is more geographically convenient and time-saving.	• Low capacity of health service provision for PLWHA in CHSCs.
			• Concerns about loss to follow-up in the referral process from CDCs to CHSCs.
Administrators and health care providers in CHSCs	Providing the HIV/AIDS case management services for PLWHA.	• Case management in CHSCs have better accessibility and integrated capacity of health care provision.	• Lack of specific funding and manpower.
			• Health care providers in CHSCs were less experienced and unstable in their position.
			• Health care providers in CHSCs have limited knowledge and skills in HIV/AIDS case management.
			• Lack of coordination and support among government sectors, hospitals, CDCs and CHSCs.
			• Discrimination against PLWHA by health care providers in CHSCS.
Managers and volunteers from CBOs	Assisting in counseling and referral of HIV/AIDS case management services.	• CBOs have good relationships with PLWHA and flexibility in working hours.	• Inadequate financial and policy support by governments.
		• CBOs can provide comprehensive counseling for PLWHA.	
PLWHA	Utilization of HIV/AIDS case management services.	• It is more convenient and accessible to utilization related health services in CHSCs.	• Fear for discrimination and lack of confidentiality when receiving health care services in local communities.
			•Fear for running into acquaintance in CHSCs.

### Participants from BH and CDCs

Most officials from BH and health professionals from CDCs favored CHSCs’ engagement in HIV/AIDS case management services. On the one hand, they considered that the increasing number of managed HIV/AIDS cases and shortage of health professionals made it difficult to improve the service quality and effectiveness. On the other hand, they thought it would be more convenient geographically and would be time-saving for PLWHA to utilize related services in CHSCs in their communities. However, their main concerns were the corresponding financial and strong policy support, CHSCs’ capacity of providing health care for PLWHA and loss to follow-up in the referral process from CDCs to CHSCs.

*“Case management services provided by CHSCs have many advantages. Firstly, it is more convenient and economical for PLWHA to come to CHSCs in such a big city. Secondly, CHSCs have both general practitioners and public health professionals who can meet the health service demands of most PLWHA.” (Male, program manager from a municipal CDC)*

*“It is necessary to transfer this service to CHSCs, but the financial support and manpower issues must be considered to ensure the service quality and sustainability of this work.” (Female, program manager from district CDC)*

### Administrators and health care providers in CHSCs

Albeit some improvement in accessibility and capacity of integrated health care provision, many CHSCs still faced difficulties after one-year implementation of HIV/AIDS case management services. Firstly, specific funding and manpower were listed as top concerns for administrators in CHSCs. They were not certain whether the services were sustainable after the program ends. Secondly, many health care providers in CHSCs were young women. They were temporarily employed but charged with several tasks. It’s hard for them to keep a stable work relationship with PLWHA. Thirdly, the health care providers had limited knowledge and skills in dealing with complicated psychological and pharmacological problems such as ART side effects. They all claimed the urgent need for professional training on case management services. Fourthly, they also concerned about the lack of coordination among government sectors, hospitals, CDCs and CHSCs in service provision, which could hardly be accomplished by CHSCs alone.

*“We could have regular interviews with most PLWHA living in our community and collect their blood sample for CD4 count test. The interview was usually arranged in our CHSC, considering the medical convenience and our personal safety.” (Female, health care provider from a CHSC)*

*“Our health providers are all part-time employers with a heavy workload. Without the program support, I am afraid this work will not be continued.” (Male, administrator from a CHSC)*

*“Most health care providers are young women and not stable in their position. The frequent labor turnover of health care provider will surely affect the follow-up intervention. Besides, our knowledge on HIV/AIDS is very limited, for example, if there is an ART side effect, we don’t know how to deal with it.” (Female, administrator from a CHSC)*

### Managers and volunteers from CBOs

Most managers and volunteers from CBOs supported this task shifting strategy. They thought that CBOs could serve as a bridge between PLWHA and CHSCs if they were given sufficient financial and policy support. Good relationships with PLWHA and flexibility in working hours were their advantages. However, they were concerned that CHSCs had inadequate skills in providing health services such as comprehensive counseling and treatment for opportunistic infections, which might cause loss to follow-up in the process of referral when PLHWA distrusted the health care providers in CHSCs. They also concerned about potential discrimination against PLWHA by some health care providers in CHSCs.

*“The resources are controlled by government and we can get little of them. We are good at HIV/AIDS-related counseling. If we assist in managing the PLWHA in CHSCs, their loss to follow-up will be very low.” (Male, CBO volunteer)*

*“There are some challenges for case management in CHSCs: the knowledge and skill of health care providers is insufficient and some of them have discriminative attitude towards PLWHA, thus PLWHA would not like to go to CHSCs.” (Male, CBO manager)*

### PLWHA

About half of the PLWHA agreed to transfer the comprehensive management services to local CHSCs, convenience was their main preference for receiving health services in CHSCs including health education and blood sample collection for CD4 count test. Nevertheless, some PLWHA were reluctant to see community doctors. They feared discrimination and lack of confidentiality when receiving health services in local communities. They were also afraid that they would run into an acquaintance in local CHSCs.

*“I received follow-up services from doctors in local CHSCs. They also held some health lectures for us. I feel it is very good and convenient here. At the beginning, I really feared that my privacy would be disclosed, but doctors were very considerate about it.” (Male, PLWHA)*

*“I usually received related health services at CDC in our district and the doctors there were very nice. But now they would transfer me to local CHSC for health services. I would be mad in that case, because my parents still don’t know my HIV positive status. In such a small area, they would inevitably know it. I would be a little better if I could choose to go to another CHSC.” (Male, PLWHA)*

## Discussion

In the present study, we used the HPT as conceptual framework to explore the challenges and enablers in the task shifting of HIV/AIDS case management services. HPT was widely used in studying HIV/AIDS prevention strategies, especially in evaluating prevention policies and human resources in areas with high HIV/AIDS prevalence [26]. The HPT framework can help both decision-makers and health care providers understand previous policy failures and plan for future policy implementations [23, 24]. Our study included key actors from different professional bodies and interest groups, especially from PLWHA groups to make the policy analysis less top-down and more evidence-based. To our knowledge, this is the first study that employed the HPT framework to assess HIV/AIDS related health services in China. As China’s HIV/AIDS response policies became increasingly information-driven [27], valuable information obtained from this study can help formulate more specific and tailored policies to guide HIV/AIDS prevention and control in urban areas.

Our study showed that shifting HIV/AIDS case management services downwards to CHSCs was feasible in terms of the policy environment, infrastructure and technical capacity of the CHSCs in urban China. Their wide distribution, technical capacity and current practice enabled them to carry out the HIV/AIDS case management services for PLWHA in local communities. Other studies have shown that community support can effectively improve PLWHA’s access to related health care services [28, 29]. Community-based case management services for PLWHA such as follow-up interventions, psychosocial counseling and education for ART adherence can improve their health outcomes, especially in a resource-constrained setting facing a shortage of health workers and/or facilities [30, 31]. Also, the increasing number of PLWHA and expansion of HIV/AIDS preventive services posed challenges to CDCs such as lack of workforce and sub-optimal quality of services. Our analysis indicated that task shifting of case management is a necessary response to the changing epidemic of HIV/AIDS in urban China. Policy makers need to ensure both political feasibility and resources accessibility to facilitate this process [32, 33].

However, the reluctant acceptance among some health care providers and PLWHA may compromise the effectiveness and sustainability of those services. Our findings indicated several challenges in the task shifting. Those challenges included lack of specific policy and stable financial support for CHSCs, inadequate manpower, relatively low capacity for health service delivery, lack of coordination among sectors, PLWHA’s fear for discrimination and privacy disclosure in local communities. As pointed out in the WHO guidelines, task shifting is not merely the move of health services from qualified health workers to less specialized health care providers, it should be implemented alongside with other efforts and resources such as financial support, skill training and supportive environment to sustain the services [11, 34].

CHSCs in urban China are mostly publicly-owned. Their funding was allocated in proportional to the number of residents in their catchment areas, whereas many PLWHA in local communities were not counted as community residents since they were migrants. Moreover, HIV/AIDS related health services were not in the priority among 11 health service tasks in the National Regulations on Basic Public Health Care, which may also discourage decision-makers from funding those services [20]. Although all CHSCs in our study were located in relatively affluent areas with well-built health service facilities and higher level of funding than the national average, financial and human resources constraint were still their top concerns for providing those case management services. As our study indicated that the transfer of certain health services should be accompanied by adequate funding. Furthermore, cultivation and cooperation with local CBOs could facilitate the task shifting process [35].

PLWHA’s concerns about confidentiality and potential discrimination were crucial factors preventing them from utilizing services at CHSCs, as was also revealed in other studies [36, 37]. Therefore, we highly recommended CDCs and CHSCs to train health care providers to improve their awareness of patients’ confidentiality and to create an anti-discrimination environment for PLWHA. Individual-based communication with PLWHA could be an appropriate way to help reduce their mistrust of community health care providers and ensure the success of task shifting process.

It should be noted that there were some limitations in this study. First, all three cities were selected from relatively affluent areas with good community health service environment, so findings of this study may reflect a higher acceptance for the task shifting than those in other urban areas of China. Secondly, actors from the national level were not included in this study, so information about how the nation-level HIV/AIDS related policies were shaped and how the program initiated was very limited. Thirdly, this study mainly focused on task shifting to community health care facilities, whereas other lay health personnel such as community health volunteers and retired nurses may also be effective shifting pathways that deserves further studies [29, 38]. Finally, evidence from one qualitative study was not sufficient for developing and modifying the task shifting policy, more quantitative research on health services are needed to comprehensively assess the impact of this task shifting strategy on the access and quality of health care for PLWHA.

## Conclusions

This study presented preliminary but informative evidence for the current policy of shifting HIV/AIDS case management services to CHSCs in urban China. Our study pointed out the feasibility and necessity of HIV/AIDS case management in terms of response to HIV/AIDS epidemic, policy environment, wide distribution and technical capacity of CHSCs. In addition, the study also suggested that this shifting strategy should be implemented with more targeted funding, planned team building, professional skill training, coordination with other sectors and education on privacy protection and non-discrimination to make the services more successful and sustainable.

## Abbreviations

PLWHA: People living with HIV/AIDS
ART: Antiretroviral Treatment
CDCs: Centers for Disease Control and Prevention
WHO: World Health Organization
CHSCs: Community Health Service Centers
HPT: Health policy triangle
FGDs: Focus group discussion
IDIs: In-depth interviews
BH: Bureau of Health
CBOs: Community-based organizations
NCAIDS: National Centers for AIDS/STD Control and Prevention
